# The Impact of the Fogarty International Clinical Scholars and Fellows Program Extends Beyond Borders

**DOI:** 10.4269/ajtmh.14-0265

**Published:** 2014-08-06

**Authors:** Catherine P. Benziger, Robert H. Gilman

**Affiliations:** University of Washington, Seattle, Washington; Johns Hopkins University, Baltimore, Maryland

The initial Fogarty International Clinical Research Scholars (FICRS) program, established in 2004, was focused on training in tropical medicine and was very successful in establishing researchers with expertise in communicable and non-communicable disease worldwide. This was a one-of-a-kind program: it allowed medical, veterinary, dental, and pre-doctoral epidemiology students from the United States to train in clinical research at sites in developing countries. The program later expanded, adding training of post-doctoral fellows (FICRS&F). It was originally administered by Vanderbilt University under a grant from the National Institutes of Health (NIH), but 2 years ago it was restructured to be administered by five University centers. As illustrated by its title, *Global Health Training for Fellows and Scholars* (GHTFS), the new program shifted emphasis from student to post-doctoral trainees.

The article by Heimburger and others,^1^ in this issue of the *AJTMH*, describes the earlier FICRS program but focuses only on a subset of the overall program: the fellows, who were either post-doctoral or post-residency. However, we believe that the most valuable part of the FICRS&F program was the training of students, which now is relegated to a minor position in the new GHTFS program.

In addition to providing funding and mentoring for 1 year of research abroad, the FICRS&F program paired each student with a young doctor or researcher who was also pursuing a career in clinical research in his/her home country.^2^ This was a major strength of the program, as it allowed United States students to be “twinned” with an in-country partner.

The selection process included an application and in-person interviews at the NIH^3^; the previous FICRS&F program started with a mandatory 2-week intensive orientation at the NIH before departure. This training was critical to the success of this program because it brought together all of the United States and foreign scholars and fellows under one roof, forming a network of “Fogarty Trainees.” Developing a mentor in global health, and being a part of the Fogarty network, was incredibly valuable in the career development of the trainees. A career in global health is often a road less traveled, especially for individuals interested in non-communicable diseases. The orientation allowed them to build relationships, foster future collaborations, and in some cases initiate life-long friendships. In addition, the meeting, which included all site mentors, was extremely useful for them to network and address common issues.

In 2012, the trainee selection and orientation process decentralized to multiple sites, called the “global health consortium,” and the FICRS&F program was eliminated. The orientation and mentor meetings were discontinued, eliminating the significant advantages previously mentioned. Our recommendation is that these meetings be reinvigorated, even if they need to be of shorter duration to address funding constraints. This would allow for the networking of trainees, the networking of mentors, and interactions between the two.

Most FICRS&F trainees did not write up their research findings until they returned to the United States. This unfortunately meant that publication was delayed or never occurred. Earlier in the program, Fogarty sites were able to provide funding for trainees to return to the international site for additional time, often more than 2 months, allowing them to focus on manuscript writing and even development of future collaborations. In addition, funds were available to present at an international meeting. We also recommend that this financial support be reinstated.

The fellows program was added to the original FICRS in 2008 to help achieve the ultimate goal: to develop independent researchers capable of obtaining independent research support. Although students were less expensive to fund than fellows, this shift was in part financially driven: the Fogarty International Center had difficulty attracting funders from NIH institutes because of the long latency period from student to NIH grantee. However, we believe this change has detracted from the main goal of the program.

In the previous FICRS&F program, in contrast to the scholars, the fellows were older (average age 33 years), more often married (64%), and currently in residency or fellowship programs (65%).^1^ Even though the fellows typically require a shorter time to becoming NIH grantees, there are downsides to focusing on this group. For the fellows, there is pressure to stay in the United States after the program as a result of restrictions on time, finances, childcare, schooling, and partner responsibilities. In our experience, many fellows go back to their previous pathway either in clinical or domestic research positions. Therefore, if the goal of the program is to develop a cohort of trained experts who work in communicable and non-communicable disease at international sites that are sustainable over time, it appears that the balance of the scholars and fellows may have then swung too far in the wrong direction. The program should equalize scholars and fellows.

To date, we have not seen any data, tabulated by year of training to correct for a latency period, of long-term success in retaining United States and international scholars or fellows in tropical medicine, either based on publications or grants in this area. The Fogarty website provides minimal information and does not provide this level of detail.^4^ We believe (and the operative word is *believe*), that more of the scholars wind up in overseas research partnerships than do the fellows. Because of their early exposure to global health research and the early stage of their careers, the scholars have more opportunity to consider their goals over the long term. We recommend that this tabulation be performed so that objective evidence is available to evaluate program success.

One other concern about both the FICRS&F and GHTFS programs is the challenge of tracking trainees from each site using the current format. The tracking system is an online database that requires trainees to tediously and annually enter all their past training, publication, and presentation information; it is not very user friendly. One recommendation is to use social media, such as Facebook, for tracking. This could be a more valuable tool to contact past trainees, update the Institute on the scholar or fellow's current activities, and facilitate tracking in the future.

Finally, what is the ultimate goal of the FICRS&F and the current GHTFS programs? The programs are currently tracking publications and grants of past trainees. However, if the goal is to develop leaders, mentors, sustainability at foreign sites, and collaborations between the United States and international researchers, an investment in both scholars and fellows, at a minimum with equal investment in both groups, is critical to the success of this program.
